# Structure-Guided Design of Peptide Inhibitors Targeting Class I Viral Fusion Proteins

**DOI:** 10.3390/pathogens15010032

**Published:** 2025-12-25

**Authors:** Narendra Kumar Gonepudi, Harry Baffour Awuah, Wang Xu, Revansiddha H. Katte, Maolin Lu

**Affiliations:** Department of Cellular and Molecular Biology, School of Medicine, University of Texas at Tyler Health Science Center, Tyler, TX 75708, USA; narendrakumar.gonepudi@uttyler.edu (N.K.G.);

**Keywords:** fusion inhibitors, peptides, class I fusion, viral fusion protein, structure

## Abstract

Viral fusion proteins are indispensable mediators of viral entry that orchestrate the fusion of viral and host membranes, making them primary targets for antiviral interventions. Class I fusion proteins, displayed on the surface of enveloped viruses (such as HIV-1, RSV, SARS-CoV-2, Nipah, influenza, and Ebola viruses), share conserved structural features, including the fusion peptide or loop and heptad repeat regions. These elements are essential for the formation of the post-fusion six-helix bundle during membrane fusion. Peptide inhibitors that mimic heptad repeat motifs have consequently emerged as an effective strategy for blocking the fusion process. This review summarizes design strategies for such inhibitors and highlights how sequence and structural insights have enabled their optimization via α-helical stabilization, hydrocarbon stapling, lactam bridges, lipid conjugation, macrocyclization, and multivalency. Using representative examples across major viral systems, this review illustrates how these strategies have led to the development of potent, stable, and even broad-spectrum antiviral peptides. This review provides insights to guide the rational design of next-generation peptide-based fusion inhibitors targeting viral membrane fusion.

## 1. Introduction

Enveloped viruses rely on membrane fusion to deliver their genomes into the host cells, a critical step in viral infection and pathogenesis. Viral fusion proteins mediate this process by undergoing extensive conformational changes that drive fusion of viral and cellular membranes (either plasma or endosomal membranes), thereby facilitating virus entry [1,2,3,4,5,6]. Besides this essential role in viral entry, fusion proteins also determine host specificity and tissue tropism and play significant roles in pathogenesis and immune evasion [1,2,3]. Their surface exposure and functional roles make them primary targets for antiviral therapeutics and vaccines. Based on their distinct structural features and modes of fusion priming and triggering, enveloped virus fusion proteins are classified into three major classes: class I, class II, and class III [1,4]. Unlike those, non-enveloped viruses such as reoviruses harbor class IV fusion proteins, also known as fusion-associated small transmembrane proteins, which act as cell–cell fusogens that merge host cells into a syncytium [7]. Class I fusion proteins are among the most extensively studied due to their critical roles in viral entry, pathogenesis, and associated mortality in pathogenic viruses, including human immunodeficiency virus type 1 (HIV-1; species *Human immunodeficiency virus 1*, genus *Lentivirus*, family *Retroviridae*), respiratory syncytial virus (RSV; species *Human orthopneumovirus*, genus *Orthopneumovirus*, family *Pneumoviridae*), severe acute respiratory syndrome coronavirus 2 (SARS-CoV-2; species *Betacoronavirus pandemicum,* genus *Betacoronavirus*, family *Coronaviridae*), nipah virus (NiV; species *Henipavirus nipahense*, genus *Henipavirus*, family *Paramyxoviridae*), Ebola virus (EBOV; species *Zaire ebolavirus*, genus *Orthoebolavirus*, family *Filoviridae*), and influenza A virus (IAV; species *Alphainfluenza virus influenzae,* genus *Alphainfluenzavirus*, family *Orthomyxoviridae*), etc. Their unique structural and mechanistic features provide a well-characterized platform for studying viral membrane fusion and guiding rational vaccine design and inhibitor development [8,9].

Class I fusion proteins, or more specifically, fusion subunits, share core structural characteristics and are generally thought to drive membrane fusion through a common mechanism. In this review, fusion proteins refer to the surface spike glycoproteins (trimers) on the viral surface, and the fusion subunits are the transmembrane subunits primarily involved in fusion. They are generally type I transmembrane proteins characterized by a trimeric α-helical coiled-coil motif and that transition from a metastable pre-fusion conformation, through multiple intermediates (plausible pre-hairpin intermediates) to a highly stable post-fusion conformation (known as the six-helix bundle—6HB) during membrane fusion [1,4,10]. Fusion proteins of this class are initially synthesized as precursor glycoproteins that undergo proteolytic cleavage by host proteases or others to form two subunits (one of which is the fusion subunit responsible for driving fusion). The fusion subunits of this class include the gp41 subunit of the HIV-1 envelope glycoprotein (Env; gp120 + gp41), the F protein of RSV and Nipah virus (F; F1 + F2), the S2 subunit of the SARS-CoV-2 spike glycoprotein (S; S1 + S2), the GP2 subunit of the Ebola virus glycoprotein (GP; GP1 + GP2), the HA2 subunit of influenza virus hemagglutinin (HA; HA1 + HA2), and others [1,4,10,11,12].

**Figure 1:**
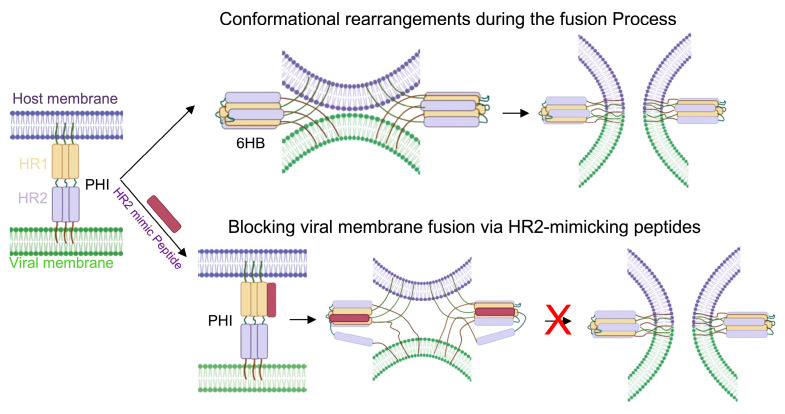
A model depicting downstream conformational transitions of the fusion protein and blockade by HR2-mimic peptides. The fusion protein undergoes a series of conformational transitions initiated by cellular cues (receptor engagement or low pH), leading to the exposure of the fusion peptide and the formation of a putative prehairpin intermediate (PHI). Subsequent folding into the six-helix bundle (6HB) drives membrane merger. HR2-mimic peptides bind to and stabilize the PHI, preventing 6HB formation and thereby blocking membrane fusion. Created in BioRender. Lu, M. (2026) https://BioRender.com/8nsohmt (24 November 2025).

**Figure 2:**
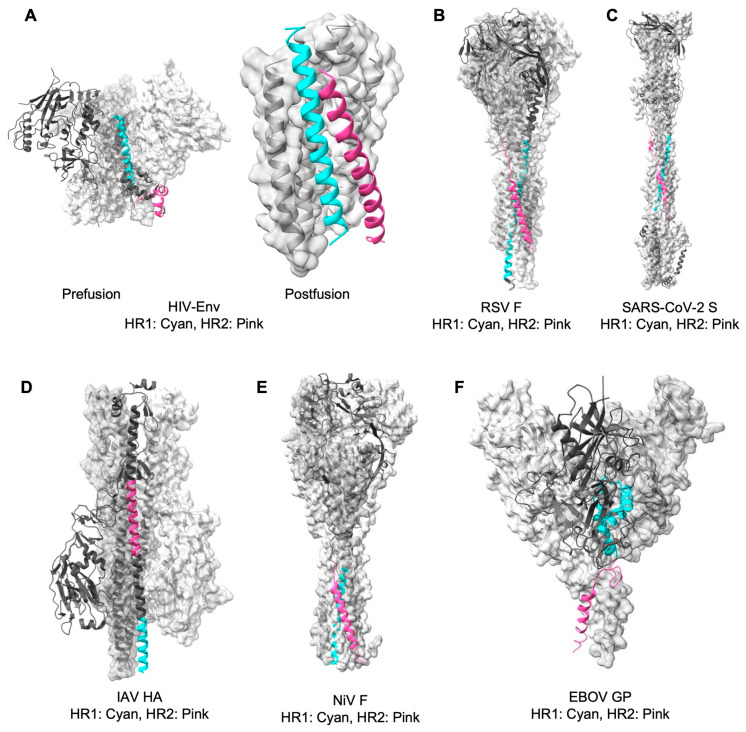
Post-fusion structures of Class I viral fusion proteins. (**A**) HIV-1 Env pre- and post-fusion protein (PDB IDs 6CM3 and AIK, respectively). (**B**–**F**) Post-fusion structures of fusion proteins for RSV, SARS-CoV-2, IAV, NiV, and EBOV ((**B**): PDB ID: 3RRR; (**C**): PDB ID: 8FDW; (**D**): PDB ID: 6Y5K; (**E**): PDB ID: 1ZTM; and (**F**): PDB ID: 5JQ3). Post-fusion of NiV F was modeled using fusion protein of HPIV3. Post-fusion structures of representative class I viral fusion proteins are illustrated as the six-helix bundle, with HR1 (shown in cyan) forming the central trimeric coiled-coil, while HR2 (shown in pink) packs against the HR1 core to complete the bundle. Peptide inhibitors typically bind to these HR1 grooves or mimic HR2 to disrupt bundle formation and block membrane fusion. Figures were generated, visualized and edited using UCSF ChimeraX (version 1.8).

Mature fusion proteins undergo subsequent conformational rearrangements in response to cellular triggers, such as receptor or co-receptor engagement or changes in endosomal pH [1,4]. These rearrangements expose the fusion peptide or fusion loop in the fusion subunits [4,11]. The fusion peptide, or fusion loop, typically a hydrophobic segment located at or near the N-terminus of the fusion subunit, is initially occluded within the pre-fusion structure and then becomes exposed and extended to insert into the host membrane, forming pre-hairpin intermediates during the fusion process (Figure 1). This is followed by the refolding of the heptad repeat regions (such as HR1 and HR2). The N-terminal heptad repeat is variously designated as NHR, HR1, or HRA, while the C-terminal heptad repeat is designated as CHR, HR2, or HRB. Throughout this review, we used HR1/HR2 nomenclature as the primary convention for consistency. These heptad repeats are stretches of amino acids with a repeating seven-residue pattern (abcdefg), where a and d are hydrophobic, promoting formation of α-helical coiled-coil structures. Refolding of these HRs results in a highly stable post-fusion conformation 6HB (Figure 1), which ultimately brings the viral and host membranes into proximity, leading to hemifusion and pore formation [1,4,13,14]. Figure 2 depicts the post-fusion structures of Class I fusion proteins, with their corresponding Protein Data Bank Identifiers (PDB IDs) indicated as follows: 6CM3 [15] and 1AIK [5] for HIV-1, 3RRR [16] for RSV, 8FDW [17] for SARS-CoV-2, 6Y5K [18] for Influenza A virus, IZTM [19] for Nipah virus, and 5JQ3 [20] for Ebola virus. The post-fusion structure of Nipah virus was modeled using the Human Parainfluenza Virus 3 [19,21]. These structures reveal atomic-level details of the conserved HR1 and HR2 motifs, although the conserved fusion peptides/loops are often unresolved. Given that interfering with HR1/HR2 interactions can prevent 6HB formation and thereby block membrane fusion [4,6,14,22,23], the 6HB architecture has served as the foundation for designing HR2-mimicking peptide inhibitors (Figure 1)—the focus of this review. Additional structural domains, such as fusion peptides/loops and other domains [24,25] (not discussed here), have also been exploited across diverse virus families, providing multiple avenues for rational inhibitor design.

Prior reviews have extensively documented the structural details of many Class I fusion proteins and their inhibition by peptide and small-molecule therapeutics [3,26,27,28,29]; however, significant knowledge gaps remain in rational design strategies, systematic comparisons of design rules and principles, and their linkage to atomic-level structural understanding, particularly for HR2-derived peptide inhibitors. In this review, we aim to fill those knowledge gaps from multiple aspects. First, we summarize the cross-viral structural conservation of Class I fusion mechanisms and systematically compare shared design principles, such as HR1 groove targeting, pocket-binding motifs, and helical stabilization, that apply across structurally distinct viruses HIV-1, RSV, SARS-CoV-2, IAV, NiV and EBOV. Second, rooted in structure-guided design strategies, we directly link atomic-level structural insights from cryo-EM and crystallography to rational peptide optimization, moving beyond empirical sequence screening. Third, we generalize design rules and principles used across various viral systems and distill the mechanistic determinants of potency, stability, and resistance resilience into actionable guidelines for future inhibitor development. Fourth, we discuss the translational and clinical implications, including the progression of peptide inhibitors from preclinical discovery through Phase II/Phase III clinical development and FDA approval. From these perspectives, we aim to integrate mechanistic understanding with therapeutic development and highlight emerging strategies for targeting conserved motifs in fusion machinery across Class I viruses.

## 2. Advances in Structural Biology of Class I Fusion Proteins

Substantial progress in structural biology has transformed our understanding of Class I fusion proteins by revealing their atomic-level structures in their prefusion, intermediate, and post-fusion conformational states. For example, early X-ray crystallography studies of influenza HA provided foundational insights by resolving both the metastable prefusion and the post-fusion structures, providing a mechanistic framework for viral membrane fusion within this protein class [6]. Similar insights were subsequently obtained for HIV-1 Env and others, revealing a highly dynamic receptor-binding and fusion-promoting structural apparatus [5,16,30,31,32,33,34]. The advent of cryo-electron microscopy (cryo-EM) has further revolutionized the field by enabling visualization of larger fusion glycoproteins, such as HIV Env, the SARS-CoV-2 spike, and EBOV GP, capturing fusion protein conformations in near-native states and during dynamic transitions [35,36,37,38,39].

These structural studies have elucidated domain organization, inter-subunit interfaces, and structural heterogeneity, and have identified conformations associated with receptor binding, antibody recognition, and fusion inhibition, providing essential guidance for vaccine and therapeutic design. For example, high-resolution structures of RSV F protein in both prefusion and post-fusion states have highlighted conformational epitopes critical for vaccine and inhibitor design [16,32,33,40]. Likewise, structural analysis of the NiV and EBOV fusion machinery uncovered unique domain arrangements and fusion-triggering mechanisms with implications for inhibitor targeting [41,42,43,44]. Structure-based design of prefusion-stabilized immunogens has been well exemplified by RSV F Hexapro, in which six proline mutations lock the RSV F protein in the prefusion state, and similar strategies have been adapted to SARS-CoV-2 S protein [45,46,47,48]. Targeting disulfide bonds and cavity-filling mutations have also been employed to improve the expression and stability of the prefusion conformation, notably useful for next-generation paramyxovirus vaccines [31,49]. Recent computational advances have further enabled de novo design and stabilization of prefusion fusion proteins, facilitating immunogen development and guiding inhibitor design with unprecedented details and efficiency [44,50,51]. By integrating structural data with molecular dynamics simulations and machine learning, researchers have modeled fusion intermediates and identified cryptic binding pockets inaccessible to typical static structural methods [52,53,54]. Collectively, these structural and computational advances have provided an atomic-level understanding of the architecture and dynamics of Class I fusion proteins, forming the basis for the rational design of vaccines, antibody therapeutics, and, most relevant to this review, peptide-based fusion inhibitors.

## 3. Overall Strategies for the Design of Peptide-Based Fusion Inhibitors

Elaborating on these concepts, a major focus has been on translating structural insights into the rational design of peptide-based fusion inhibitors. Most structural studies have focused on characterizing or stabilizing pre-fusion structures, as discussed above, and sometimes conformational intermediates [55,56] during membrane fusion. Nevertheless, the hydrophobic grooves on the HR1 trimeric coiled coil, revealed in high-resolution structures of post-fusion 6HB, actually serve as the key target for designing HR2-mimicking peptide inhibitors (Figure 2) [57,58]. Structural information extending beyond HR regions has also enabled the design of peptides targeting allosteric sites to stabilize prefusion conformations or restrict conformational flexibility [23,57,58,59]. Overall, structure-guided design of peptide inhibitors aims to arrest the fusion cascade at intermediate stages, between prefusion and post-fusion, by mimicking native interaction domains or blocking critical protein-protein interfaces, particularly the HR1-HR2 interaction that forms the 6HB (Figure 2) [23,60,61]. Representative inhibitors, such as HIV-1 enfuvirtide (T20) [62,63,64,65] and LP-98 [66], effectively inhibit fusion and exert potent antiviral effects.

**Figure 3:**
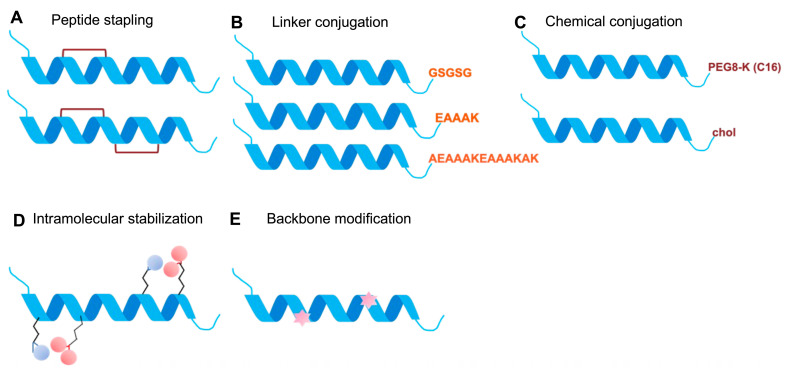
Design strategies for peptide-based antiviral inhibitors. (**A**) Peptide stapling to stabilize α-helical structure, enhances target affinity, and improves proteolytic resistance. (**B**) Conformational constraint strategies using flexible (linear) linkers versus rigid “molecular braces” to modulate peptide geometry and binding. (**C**) Chemical conjugation approaches, including PEGylation to improve solubility and serum half-life, and cholesterol tagging to enhance membrane association and cellular uptake. (**D**) Introduce intramolecular crosslinks that stabilize secondary structure and increase resistance to degradation. Colored balls indicate reactive functional groups for cross-linking. (**E**) Backbone modifications, such as N-methylation and peptidomimetics, to improve stability, bioavailability, and pharmacokinetics. Stars indicate places where residue side chains (or termini) are altered.

**Figure 4:**
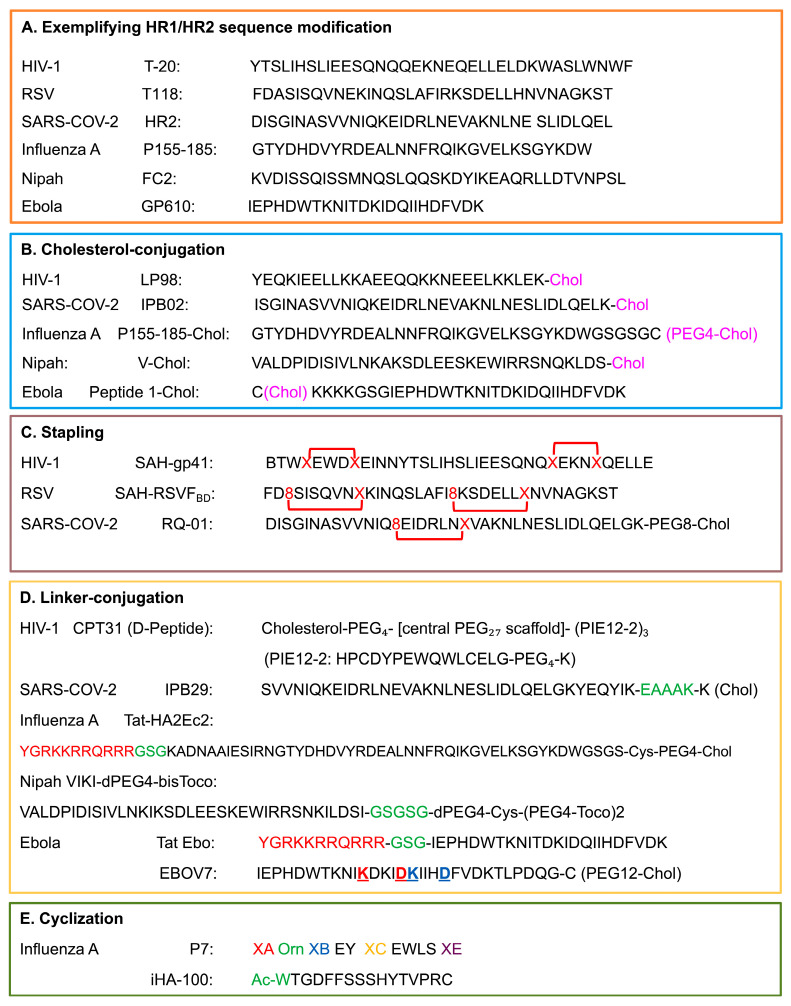
Representative peptide inhibitors targeting Class I viral fusion proteins, grouped by major modification strategies. (**A**) HR1/HR2 sequence modifications: Native peptides derived from heptad-repeat regions, and sequence-optimized HR1/HR2 mimetics. (**B**) Cholesterol-conjugated peptides: Lipid-modified inhibitors designed to increase membrane anchoring, local concentration at fusion sites, and antiviral activity. Chol, Cholesterol; PEG, polyethylene glycol. (**C**) Stapled peptides: Hydrocarbon-stapled helices that stabilize α-helical structure, improve protease resistance, and strengthen HR1/HR2 binding (8 = R-octenyl-alanine; X = S-pentenyl alanine). (**D**) Linker-conjugated peptides: Inhibitors incorporating flexible or rigid linkers to modulate peptide conformation or spacing for Chol conjugation for enhanced target engagement. Underlined residues are those engaged in a side-chain-to-side-chain lactam. The two consecutive lactam bridges are indicated in red and blue, respectively. (**E**) Cyclized peptides: Backbone- or side-chain-cyclized inhibitors that improve structural rigidity, binding affinity, and resistance to degradation. XA, 5-phenyl-norvaline; Orn, Ornithine; XB, N-methylated leucine analog; XC, di-chlorophenyl alanine; XE, N-methylated amino acid variant; Ac-W, N-(2-chloroacetyl) tryptophan.

Built upon HR1/HR2 interactions, a range of design strategies for peptide-based fusion inhibitors (Figure 3 and Figure 4) have been developed to improve α-helical stability, membrane permeability, interface complementarity, potency, and resistance resilience. Detailed atomic models have identified flexible or solvent-exposed residues suitable for chemical modifications, such as hydrocarbon stapling, lipid conjugation, and incorporation of noncanonical amino acids, that enhance α-helicity, proteolytic resistance, and membrane permeability [67,68,69,70,71,72]. These design strategies (Figure 3) demonstrated with examples (Figure 4) follow common principles: (1) achieving strong interface complementarity to HR1 grooves, (2) incorporating pocket engaging and electrostatic motifs to enhance affinity, (3) stabilizing peptide conformation through hydrocarbon stapling, lactam bridges or cyclization, (4) using membrane-targeting elements, such as cholesterol conjugation and optimized linkers, to concentrate inhibitors at the fusion interface and support endosomal activity where required (e.g., EBOV and IAV), and (5) additional engineering strategies, including dimerization, tandem architectures, heterotypic multivalency and backbone modification with non-canonical residues, D-chirality, and peptidomimetics. All these approaches have guided the optimization of peptide-based fusion inhibitors, as exemplified across different class I viral fusion proteins (Figure 4), to achieve broad coverage and decouple pharmacokinetics from natural peptide constraints. We will exemplify these design approaches and principles in the following section using representative Class I viral systems and peptide inhibitors (Figure 4).

## 4. Representative Peptide Inhibitors Targeting Class I Viral Fusion Proteins

### 4.1. HIV-1 Env Fusion Subunit gp41

HIV-1 entry into host cells involves sequential binding of the envelope glycoprotein surface subunit gp120 to the host cell receptor CD4 and a coreceptor (CXCR4/CCR5), triggering conformational rearrangements in Env that expose the fusion subunit gp41 [4,13,73,74]. The gp41 fusion peptide then inserts into the host membrane and subsequently folds back on the C-terminal heptad repeat (HR) to form the 6HB: (HR1/HR2)_3_, driving membrane fusion [4,13,73]. This multistep mechanism established gp41 as a critical therapeutic target for blocking fusion. Currently, most peptide fusion inhibitors are derived from the gp41 HR1 and HR2 domains, especially HR2 [75]. Early studies identified CS3, a peptide derived from the HR2 domain, prompting the development of additional gp41-derived fusion inhibitors, which, in turn, led to the generation and clinical approval of T20 [63,64,76,77]. Of note, structural characterization of gp41 revealed the canonical 6HB architecture, in which three HR2 helices pack antiparallel around a central HR1 coiled coil [78,79,80]. The discovery of a deep hydrophobic pocket at the C-terminal end of HR1 provided a structural basis for rational inhibitor design, enabling peptide derivatives with markedly enhanced binding affinity (e.g., C34, Kd = 0.0007 nM vs. T20, Kd = 30 nM) [81].

T20 in HIV-1 was the first proof-of-concept with clinical success for HR2-mimicking peptide inhibitors that block 6HB formation by preoccupying HR1 binding sites [62,63]. Derived from the HR2 region (amino acids 638–673), T20 (Figure 4) contains an HR-binding domain (residues 638–666) that interacts with HR1, and a tryptophan-rich membrane-binding domain (residues 666–673) [82]. Despite clinical validation, T20 exhibits moderate potency (IC_50_ ~3 nM), rapid emergence of drug-resistant variants, a short plasma half-life, and notable adverse effects, such as injection-site reactions, peripheral neuropathy, etc., which ultimately lead to its discontinuation [28,83]. These shortcomings motivated the development of next-generation peptides. C34, a 34-residue peptide that engages the conserved HR1 hydrophobic pocket via its pocket-binding domain, achieves sub-nanomolar potency, remarkably improved binding affinity (Kd = 0.0007 nM vs. 30 nM for T20), and improved resistance profile [5,84].

Second-generation inhibitors, such as T-1249 (tifuvirtide), combine pocket-binding features with extended HR sequences (C-terminal tryptophan-rich motif). Incorporating sequences from HIV-1, HIV-2, and SIV, T-1249 showed enhanced potency and activities against T20-resistant strains [85,86,87,88]. Meanwhile, efforts to introduce electrostatic stabilizing motifs (e.g., Glu–Arg ER pairs) and hydrocarbon stapling (SAH-gp41) (Figure 4) [89], increased 6-HB stability and plasma half-life [90,91]. Conjugating peptides to serum albumin to increase the plasma half-life through peptide residue modification with 3-Maleimidopropionic acid resulted in the design of albuvirtide (FB0006) [92]. Efforts in detailed structure-activity refinement (SAR) further defined key structural determinants of activity, including the pocket-binding motif, helical stabilization requirements, and membrane-interaction elements [86,93]. Building on these principles, HP23, a rationally designed 23-residue peptide featuring an M-T hook structure [94], demonstrated exceptional potency and retained activity against resistant mutants [94,95,96,97,98]. This design strategy was also applied in the development of 2P23 and its derivatives [98].

Lipid conjugation represented another milestone in enhancing peptide performance. Cholesterol conjugation significantly increased membrane affinity and stability, thereby establishing the membrane-targeting paradigm, as exemplified by C34-Chol, which exhibited 25–100-fold greater potency than C34 [99,100,101]. A series of optimized lipopeptides, including LP-11, LP-19, LP-40, LP-46, LP-80, and LP-98 (Figure 4), further improved potency into the picomolar range through lipid (fatty acids, cholesterol, sphingolipids, etc.) conjugation and refined linker chemistry [102]. Among them, LP-98, currently in Phase II clinical trials, is the latest generation. Structural characterization revealed that LP-98 achieves exceptional potency through optimal clustering of hydrophobic residues, a network of 15 hydrogen bonds, two electrostatic interactions, and a salt bridge at the 6HB interface [66].

Alternative conjugation approaches such as PEGylation have also been explored to improve peptide stability and solubility. N-terminal PEGylation of C34 (2 kDa and 5 kDa) significantly extended proteolytic stability while maintaining reasonable inhibitory activity [103,104]. Recent innovations have combined peptide fusion inhibitors with entry-targeting antibodies, such as 2P23-PRO140, a fusion construct that simultaneously targets CCR5 (via the PRO140 antibody) and gp41 (via the 2P23 peptide) [105]. Dimerization of HR2 peptides, especially cholesterol-conjugated dimers, has also been shown to significantly enhance antiviral activity by increasing local concentration and binding avidity [71]. Leveraging upon the enhanced potency achieved through HR2 dimerization strategies, next-generation approaches have enabled the use of D-peptide fusion inhibitor technology to address susceptibility by host L-proteases. CPT31 (Figure 4), a D-peptide (trimer of PIE-12-2 with cholesterol moiety), exemplifies this approach. It has demonstrated potent antiviral efficacy in both in vitro and in vivo models and advanced to Phase I clinical evaluation [106].

Beyond the well-characterized HR2-mediated inhibition pathway, HR1-derived fusion inhibitors have emerged as an alternative therapeutic approach, distinguished by their ability to disrupt the endogenous HR1 coiled-coil architecture itself rather than competitively blocking HR2 binding. N36, a prototypical 36-residue HR1-derived peptide [107,108,109], demonstrated poor potency as a monomer (IC_50_ ~16 μM) due to aggregation [110,111] and mixed oligomerization states [112], making it 300–500 fold less potent than HR2 peptides like C34 [109]. Engineering N36 into stable trimers via fusion to the GCN4-IQ coiled-coil domain (yielding IZN36) [110] achieves ~500-fold enhancement in anti-fusion activity against R5-tropic viruses through stabilized trimeric assembly and improved HR2 binding [112,113].

HIV-1 fusion inhibitor optimization (Table 1) follows a defined hierarchy, with strong HR1 pocket engagement preceding membrane anchoring, reflecting the unique fusion mechanism and structural constraints of Env [114,115]. Structure-guided targeting of the conserved hydrophobic HR1 pocket is critical for high-affinity binding, as exemplified by the HR2-derived peptide C34, which exhibits ~43,000-fold higher binding affinity than T20, yet only ~10-fold greater cellular antiviral potency, indicating that binding affinity and functional activity are not directly proportional. This principle guided the design of subsequent inhibitors, such as HP23, which incorporates an M–T hook motif, and LP-98, which forms extensive hydrogen-bonding and electrostatic interactions. Membrane anchoring, typically achieved via cholesterol conjugation, further enhances antiviral potency 25–100-fold by concentrating inhibitors at lipid-rich fusion sites. Refinement of linker flexibility and lipid optimization (cholesterol vs. fatty acids vs. sphingolipids) has enabled additional gains in activity, with LP-98 achieving sub-picomolar potency.

### 4.2. RSV F Protein

Similarly to HIV-1 Env, the F protein of RSV is metastable and undergoes spontaneous or stimulus-induced conformational changes from a prefusion to post-fusion form [4]. Unlike the predominantly α-helical HIV-1 gp41, RSV F features a shorter six-helix bundle, distinct heptad repeat patterns, and a β-sheet propensity in the HR2 region [33,116]. The HR1 domain forms a stable trimeric coiled coil interacting with HR2 to form the critical 3:3 hexamer 6HB [117]. The structural insights into RSV F have been pivotal in guiding structure-based peptide inhibitor design aimed at blocking 6HB formation [61,118,119].

Early RSV heptad repeat (HR1/HR2) derived peptides were designed after the HIV paradigm but displayed only micromolar or sub-micromolar potency [120,121]. Among those, peptides derived from the HR2 region of RSV F (residues ~485–524) have been most extensively studied [29,32,122]. One of the earliest, T-118 (Figure 4) [123], demonstrated inhibitory activity with an IC_50_ of approximately 1500 nM in cytopathic effect assays and ~3500 nM in cell-fusion assays, indicating feasibility but limited potency [32,123,124]. Subsequent structure-activity relationship studies revealed that both peptide length and sequence strongly affect potency. Longer peptides offered broader binding interfaces and improved stability. C20 (20 aa, residues 492–511) and C30 (30 aa, residues 482–511) peptides achieved IC_50_ of 14.9 μM and 6.8 μM, respectively, in contrast to the lack of activity of C17 (17 aa, residues 495–511) at 100 μM. Further optimization led to the F478–516 peptide spanning the broader portion of the HR2 domain, which exhibited activity in the low micromolar range, i.e., ~1–10 μM, [122,125].

To enhance stability and binding affinity, several engineered poly-peptides containing alternating HR1 and HR2 sequences were developed, including 5-Helix, HR121, and HR212 constructs [126,127,128]. These multimeric designs improve both structural integrity and inhibitory activity. For example, the 5-Helix construct consisting of three HR1 helices (N57) and two HR2 helices (C49) alternately linked by flexible peptide linkers achieved an IC_50_ of 3.36 ± 0.23 μM, compared with 3.74 ± 0.67 μM and 7.95 ± 1.01 μM for HR121 and HR212, respectively [32,126]. Despite structural superiorities, the IC_50_ values of these first-generation multimeric RSV constructs (~3–8 µM) did not drastically exceed F478–516, likely due to steric bulk hindering access to the crowded fusion site or non-optimized interface contacts in the chimera. However, they represent a more robust structural scaffold for further optimization than linear peptides.

A major advancement came with hydrocarbon-stapled peptides (Figure 4), which stabilize α-helical conformations by covalently linking side chains to generate “staples.” These modifications substantially improve helicity, proteolytic resistance, and cellular uptake [126,129,130]. Single-stapled constructs provided limited inhibition; however, double-stapled variants exhibited nanomolar activity in HEp-2 cells [89,130,131]. Among these, the stabilized α-helix of the RSV F peptide, SAH-RSVF_BD_ (Figure 4), is the best-characterized one [131]. This 35-residue double-stapled peptide derived from RSV HR2 displayed markedly enhanced structural stability and antiviral potency compared to its unmodified counterpart, validating the stapling approach for Class I fusion systems [131]. Gaillard and colleagues (2017) further introduced the “peptide 4 series”, a group of short (20-mer) double-stapled constructs with tighter staple spacing. Variants such as peptide 4ca exhibited remarkable potency, achieving superior antiviral activity and greater resistance to proteolytic degradation compared with longer stapled peptides. Intranasal administration of peptide 4ca substantially reduced RSV replication in the upper and lower respiratory tracts of mice, establishing proof of concept for clinical translation [131,132].

Persistent viral evolution has also driven resistance-driven innovation. Common escape variants such as K394R and D489Y prompted the design of novel candidates like CL-A3–7, which targets the F-IGF1R interface rather than the internal fusion machinery [133]. This structure-based redirection of focus toward receptor-associated interactions offers a promising route to overcoming traditional resistance mechanisms.

The success of these engineered RSV F inhibitors, along with the previously discussed HIV-1 gp41 examples, highlights the versatility of structure-guided peptide design strategies, which have since been extended to other Class I fusion systems, such as the SARS-CoV-2 S2 protein discussed next.

Altogether, advances in peptide design targeting RSV F have followed a progressive refinement of structural and functional principles (Table 2). T118 functions as a linear HR2-mimetic peptide that binds the HR1 region with moderate potency and stability. Interface optimization via sequence extension or multimeric scaffolds, enhanced HR1 engagement and neutralization potency, as seen in C30, F478–516, and 5-Helix peptides. SAH-RSVF_BD_ and 4ca incorporated hydrocarbon stapling to stabilize the α-helical conformation, preserving a pre-fusion-like structure and enhancing proteolytic resistance and binding affinity. These design strategies illustrate a rational trajectory from simple sequence mimicry to interface optimization, to structural stabilization, demonstrating a roadmap for improving potency, stability, and breadth in RSV F-targeted peptide therapeutics.

### 4.3. SARS-CoV-2 S Protein S2 Subunit

The spike protein (S, S1 + S2) of SARS-CoV-2 consists of two subunits, in which S2 drives membrane fusion after the S1 dissociates following ACE2 binding and proteolytic cleavage at the S2’ site [134,135]. Structural rearrangements in S2 include key steps, such as fusion peptide exposure, insertion into the host membrane to form putative pre-hairpin intermediates, and the folding of HR1 and HR2 back onto each other to form a 6HB [4,134]. The pre-hairpin intermediate is thought to adopt a configuration in which HR1 trimerizes and exposes hydrophobic grooves before HR2 folds back. This extended intermediate creates a therapeutic window for HR2-derived peptides to bind competitively and block fusion [4,134,136].

Prior to the SARS-CoV-2 pandemic, EK1 emerged as a pan-coronavirus inhibitor, rationally designed from HCoV-OC43 HR2, and demonstrated broader activity against SARS-CoV, MERS-CoV, etc. [137]. When SARS-CoV-2 emerged, EK1 showed an IC_50_ of 315 nM for cell–cell fusion assays and 2468 nM for virus infection [137,138]. IPB01, designed from the SARS-CoV-2 native HR2 sequence (residues 1168–1203, Figure 4), potently inhibited cell–cell fusion (IC_50_ = 22 nM) but displayed weak activity against pseudovirus and authentic viruses (IC_50_ > 33 μM), which highlights the critical importance of membrane anchoring for effective viral entry inhibition [137,139].

Cholesterol conjugation, as a lipidation strategy, was then used to improve the antiviral activity of peptides. For example, the next generation EK1C4, in which EK1 was linked via PEG4 spacer to cholesterol, achieved a 242-fold increased potency for fusion (IC_50_ = 1.3 nM vs. 315 nM) and 68-fold improvement against authentic virus (IC_50_ = 36.5 nM vs. 2468 nM) [140]. Structures of EK1 in complex with SARS-CoV-2 HR1 linked the better performance to key binding determinants, such as enhanced hydrophobic interactions (L10, F9, V7 replacing smaller residues in native HR2), optimized salt bridges (E21 to K25, K18), and critical pocket-binding residues (Y30) [137]. Computational approaches identified specific mutations (EK1-8F, EK1-8Y, EK1-29A) that increase binding affinity by enhancing HR1 contacts and expanding the interface area [141]. Another notable example is IPB02 (Figure 4), the cholesterol-conjugated IPB01, which demonstrated IC_50_ values of 25 nM for fusion and 80 nM for pseudovirus infection, showing improved efficacy compared to unconjugated IPB01 and exhibiting cross-inhibitory activity against HIV-1, HIV-2, and Simian immunodeficiency virus (SIV) [139]. The lipidation strategy pioneered by EK1C4 and IPB02 directly informed IPB29 (Figure 4), which is now in Phase III clinical trials in China. IPB29 incorporates an EAAAK rigid linker between the HR2 peptide and cholesterol moiety, further enhancing α-helical stability and binding affinity [142,143].

[SARS_HRC_-PEG_4_]_2_-Chol, a dimeric lipopeptide with two HR2-derived chains linked to a single cholesterol anchor, achieved IC_90_ ~35 nM with biodistribution studies showing prolonged lung tissue retention compared to monomeric peptides [144,145]. In addition, the stapled approach was also applied, exemplified by the M2PA peptide, a short, double-hydrocarbon-stapled α-helical peptide derived from the HR2 region [146].

Lipopeptides and stapled peptides were further optimized after more confined S2 elements were identified. Structural analysis showed that HR2 can extend beyond the traditional core, with N-terminal residues 1159–1179 forming structured motifs making extensive contact with HR1 grooves [46,144,146,147,148]. This insight prompted the design of P42 (spanning residues 1162–1207) with six additional N-terminal residues, which exhibited an IC_50_ of ~5 nM [148]. Its derivative, P40-LP, containing four extended N-terminal residues (VDLG) plus cholesterol conjugation, demonstrated potent broad-spectrum activity against SARS-CoV-2 variants, including all Omicron sublineages and other human coronaviruses. P40-LP showed synergistic effects when combined with IPB24 (a C-terminal extended peptide), suggesting that peptides targeting distinct HR1 groove regions can cooperatively enhance inhibition [147]. Later, combinatorial strategies like stapled lipopeptides incorporating hydrocarbon crosslinks to rigidify HR2 helices combined with cholesterol anchoring, led to the development of RQ-01, which achieved IC_50_ of 3.4–8.9 nM against Omicron variants and was progressed to Phase II clinical trials [149,150]. To address peptide degradation limitations while maintaining HR1-binding pharmacophores, peptidomimetics like XY4-C7, a sulfonyl-γ-AApeptide-PEG24-Chol, using an unnatural backbone to mimic HR2, demonstrated potent in vitro and in vivo activity with protease resistance [151]. These advanced design strategies have been integrated into clinical candidates like YKYY017, which progressed to Phase II trials in 2025 [152]. Unlike the HR2-derived inhibitors, a 19-mer derived from the internal fusion peptide region, PN19, exhibited potent inhibitory activity against SARS-CoV-2 variants with no cytotoxicity [153].

All the above examples illustrate the key design rules (Table 3) that relate to generic strategies, such as EK1 (HR2 sequences from HCoV-OC43, MERS-CoV, and SARS-CoV-2) that emerged as a pan-coronavirus fusion inhibitor, to competitively bind HR1 and block 6HB formation. Derivatives such as EK1C4 and IPB02 incorporated cholesterol conjugation via PEG_4_ spacers, anchoring peptides to fusion-active membranes, and achieved dramatic potency gains 68–242-fold. Structural optimization, including hydrophobic substitutions (L10, F9, V7), salt bridges (E21-K25, K18), and critical pocket-binding residues (Y30), enhanced HR1 interactions, while computationally guided mutations (EK1-8F, EK1-8Y, EK1-29A) further improved potency. IPB29, advancing to Phase III trials, introduced an EAAAK rigid linker for α-helical stabilization and membrane anchoring, complemented by dimerization ([SARSHRC-PEG_4_]_2_-Chol) and N-terminal sequence extensions (P42, P40-LP) to exploit expanded HR1 grooves. More recently, stapled lipopeptides combining hydrocarbon crosslinking with cholesterol anchoring (RQ-01) achieved low-nanomolar activity against Omicron variants and progressed to Phase II trials. Collectively, these strategies illustrate a progressive, structure-guided design trajectory from HR2 mimicry to lipidation, helical stabilization, and interface optimization, enabling potent, durable, and broadly active SARS-CoV-2 fusion inhibitors.

### 4.4. Human Influenza Virus Hemagglutinin HA

Influenza viruses are classified into four main types: A, B, C, and D, of which A, B, and C are known to cause illness in humans [154]. The viral envelope contains two critical glycoproteins: hemagglutinin (HA) that mediates cell attachment and fusion, and neuraminidase (NA) that facilitates virion release. Influenza virus type A is further classified into subtypes based on HA (H1–H18) and NA combinations (N1–N11), of which, H1, H2, H5, H6, H8, H9, H11–H13, H16–H18 (phylogenetically similar stem epitope) were placed in group 1 and H3, H4, H7, H10, H14, H15 (phylogenetically distinct stem structure) were placed in group 2 [4,155]. The binding of the HA of the Influenza virus to the host cell via receptor (s) (sialic acid) mediates endocytosis, leading to the entry of the virus into the host cell. Later, fusion is triggered by low pH in endosomes, causing the fusion peptide containing hemagglutinin (HA2) to undergo conformational changes that drive fusion [156,157]. The structural complexity of HA, with its receptor-binding head domain and pH-dependent fusion mechanism, presents unique challenges for antiviral targeting. However, the HR2 is well-conserved, and stem regions also contain conserved epitopes recognized by broadly neutralizing antibodies [158,159], making them attractive targets for the design of peptide-based inhibitors.

Linear peptides derived from the HA2 fusion peptide or heptad repeat regions, however, exhibited weak micromolar potency, high proteolytic susceptibility, and poor pharmacokinetics [26,160]. To enhance membrane association and cellular uptake for endosomal targeting, cholesterol conjugation was applied to improve HA2-derived peptides, such as P155-185-chol (targeting H3N2, Figure 4) [56,161,162] and C20-Jp-Hp. C20-Jp-Hp is a hybrid peptide created by conjugating two short antiviral peptides, i.e., Jp and Hp with a C20 lipid chain [160,163,164]. This peptide exhibited an IC_50_ of 0.53 μM against H1N1 (A/Puerto Rico/8/34) with broad activity across H1N1, H3N2, drug-resistant NA-H274Y mutants, and influenza B viruses (IC_50_ 0.5–2.0 μM) [163,164]. To further improve the intracellular localization of the peptide, a cell-penetrating peptide sequence derived from HIV-1 TAT was combined with a lipid moiety (Cholesterol in Tat-HA2Ec2 in Figure 4 and Tocopherol in Tat-HA2Ec3), which has been shown to enhance both in vitro and in vivo efficacy [165].

High-resolution structures of antibody-complexed HA revealed that the conserved stem epitope is recognized primarily by the heavy chain complementarity-determining region 3 (HCDR3) loops and framework region 3 (FR3) residues [166]. Kadam and his team then pioneered the design of cyclic peptides based on HCDR3 of FI6v3 and FR3 of CR9114, which led to the development of the P series (P2, P3, P4, P5, P6, and P7, Figure 4) with approximately 100-fold affinity improvement through constraining the structure via rational cyclization and incorporating non-proteinogenic amino acids [166,167,168]. Later, macrocyclic peptides with bifunctional inhibition (inhibiting both HA-mediated viral adsorption to cells and membrane fusion) were developed using the Random non-standard Peptides Integrated Discovery (RaPID) platform, resulting in the generation of iHA-100 (Figure 4) with broad group 1 activity against H1N1, H5N1, H2N2, and H6N1 [169].

Antibody hotspot mapping and computational docking against conserved epitopes of the HA stem further enabled the design of D-peptide inhibitors (mirror-image coordinates) to overcome protease resistance conferred by L-proteases [170]. In contrast, the Frog defensin basic peptide (FBP) represents a nature-inspired optimization strategy derived from the naturally occurring frog defensin Urumin [171]. Later, it was optimized through charge modification to broaden its antiviral spectrum against influenza A and B viruses by binding to HA and blocking the low-pH-induced conformational changes required for HA-mediated fusion in the endosome [171,172].

Key design rules from Influenza A anti-HA peptide inhibitors (Table 4) include lipid conjugation (e.g., cholesterol in P155-185-chol and Tat-HA2Ec2/3 for endosomal targeting and improved pharmacokinetics), structural constraint via cyclization/stapling (e.g., P series yielding ~100-fold affinity gains against group 1 HA), and sequence optimization with non-natural amino acids (e.g., RaPID-derived iHA-100 for broad H1N1/H5N1 activity) which directly align with generic strategies like HR2 mimicry, chemical modifications for stability, and enhanced delivery. These optimizations translate micromolar limits of linear peptides into nanomolar potency across subtypes. These advancements exemplify broader structure-based principles for pan-influenza virus therapeutics targeting conserved HA fusion machinery.

### 4.5. Nipah Virus (NiV) F Protein

The fusion (F) protein of the Nipah virus (NiV) mediates viral and host cell membrane fusion through a mechanism characteristic of Class I viral fusion proteins, though with distinct molecular features. The F protein undergoes proteolytic activation in endosomes, exposing the hydrophobic fusion peptide for membrane insertion. Upon receptor binding by the attachment protein NiV-G to ephrinB2 or ephrinB3, conformational changes in NiV-F are triggered, leading to refolding of HR1 and HR2 into a stable 6HB that drives fusion of viral and host cell membranes [4,173]. Peptides, derived from the C-terminal heptad repeat (HR2) region of NiV F protein, mimic the HR regions and bind to HR1, therefore preventing the formation of the post-fusion conformation. The 42-mer peptide was designed based on the HR2 sequence (residues 447–489) [174,175]. Longer peptides like this, although having higher binding affinity, may suffer from poor solubility, low synthetic yield, and high toxicity. This peptide was thus further optimized into 36-mer versions with chemical modifications (e.g., capping, PEGylation and cholesterol conjugation). These modified peptides include capped NiV FC2 peptide (Figure 4), N-PEG-NiV FC2 peptide, and C-PEG-NiV FC2 peptide, among which the N-PEG-NiV FC2 peptide was most potent [174,175] and V-Chol (Figure 4) [176].

N42NiV/HeV(L6)C32HPIV3 is a chimeric fusion core peptide construct designed to inhibit Nipah virus (NiV) and Hendra virus (HeV) fusion by targeting the HR1 (N42) region of NiV/HeV and the HR2 (C32) region of human parainfluenza virus 3 (HPIV3) joined by a short linker (L6). The high-resolution structure of this peptide revealed the molecular determinants underlying heterotypic peptide superiority. Enhanced binding was attributed to optimized interhelical packing and hydrophobic interactions within key groove regions [176]. Guided by these insights, the HPIV3 HR2 peptide was engineered with the following specific substitutions: Glu459 to Valine, Ala463 to isoleucine, Gln479 to lysine, and Lys480 to isoleucine, and conjugated to lipid moieties, resulting in VIKI-dPEG4-chol and VIKI-dPEG4-bisToco (Figure 4) [177]. These modifications yielded potent inhibitors with an IC_50_ of approximately 1–7 nM [176] likely reflecting synergistic effects from both the sequence optimization and the membrane-targeting effect of cholesterol conjugation [178].

More recently, stapled lipopeptides have shown even greater potential, exhibiting nanomolar-range inhibition against RSV, Ebola virus, and NiV. These peptides display enhanced protease stability and cellular permeability. Preliminary clinical studies of stapled lipopeptides (RQ-01, Figure 4) targeting SARS-CoV-2 support their feasibility as broad-spectrum antivirals with potential applicability to NiV and other highly pathogenic viruses [150].

Notable design rules for NiV F protein peptide inhibitors (Table 5) align with generic solubilization strategies. Common strategies include shortening long HR2 peptides (e.g., 42-mer to 36-mer) and applying modifications such as N-terminal PEGylation (e.g., N-PEG-NiV FC2) or capping to enhance synthetic yield, solubility, and reduce toxicity while retaining HR1 binding affinity. The use of structure-guided heterotypic optimization, such as chimeric constructs (e.g., N42NiV/HeV(L6)C32HPIV3) or targeted substitutions in HPIV3 HR2 (Glu459Val, Ala463Ile, Gln479Lys, Lys480Ile for VIKI peptides), effectively improves interhelical packing and hydrophobic interactions, exemplifying chimeric sequence engineering. Integrating lipidation (e.g., cholesterol via PEG linkers in VIKI-dPEG4-chol) with stabilization (e.g., hydrocarbon stapling in RQ-01 lipopeptides) can boost membrane targeting, protease resistance, permeability, and broad-spectrum nanomolar potency.

### 4.6. Ebola Virus (EBOV) GP

The fusion of the Ebola virus (EBOV) with host cells is mediated by its envelope glycoprotein (GP), which consists of two subunits: GP1 and GP2 [179,180]. Among these, GP2 serves as a key target for peptide-based inhibitor design [34,180,181]. Structural rearrangements within GP during the fusion process form the foundation for rational inhibitor development. After endocytosis, the GP1 subunit undergoes proteolytic cleavage by cathepsins within acidic endosomes, thereby exposing the Niemann-Pick C1 (NPC1) receptor-binding site [182,183]. Binding of NPC1 to cleaved GP1 triggers conformational changes in the GP2 subunit, which contains an internal fusion loop that inserts into the host endosomal membrane [183]. GP2 subsequently refolds its heptad repeats into the 6HB, bringing the viral and endosomal membranes into close apposition to facilitate membrane fusion and release of the viral genome into the cytoplasm [179,182]. The transmembrane domain and fusion loop cooperate to open a fusion pore, completing the fusion process inside the late endosome [4,183,184,185].

Because EBOV fusion occurs in late endosomal compartments, a less accessible target site compared to HIV-1, it poses additional challenges for peptide targeting. Nevertheless, peptides directed against HR2 such as EBOV GP610 (Figure 4) [186], have demonstrated inhibition of EBOV pseudovirus infection [181,186]. Endosomal targeting has been achieved by conjugating the HR2 EBOV peptide to either the arginine-rich segment of the HIV-1 Tat protein, known for its endosomal localization, i.e., Tat Ebo (Figure 4) [181], or to a cholesterol group, i.e., Peptide 1-Chol (Figure 4) [187,188]. Using a multilayered engineering strategy, Pessi et al. designed a 30-mer peptide that integrates multiple optimization strategies, including cholesterol conjugation (via cysteine residues at C-terminus), PEGylation (PEG12 as spacer), helix stapling through Lys(i)–Asp(i + 4) lactam bridges, and sequence extensions at both termini [189]. Among the peptides generated, EBOV7 (Figure 4) showed the greatest potency, with an IC_50_ of 0.5 µM, and demonstrated 100% survival in mouse models when administered subcutaneously for 9 days [189].

Inspired by successful HIV-1 HR1 engineering, additional efforts led to the development of eboIZN39IQ, a chimeric peptide that embeds coiled-coil modules (IZ and IQ) at the C-terminus of the natural EBOV HR1 sequence. Binding analysis demonstrated a Kd of 14 nM for eboIZN39IQ and its target GP2, comparable to the binding affinities observed for HIV-1 HR1-peptides and their cognate targets [149,190].

Collectively, design rules for EBOV GP2-targeted peptide inhibitors (Table 6) emphasize multivalent modifications for endosomal access, such as cholesterol conjugation via C-terminal cysteine with PEG12 spacers in EBOV7, which overcomes late endosome barriers to achieve 0.5 µM IC_50_ and full mouse survival, demonstrating lipidation for membrane anchoring and intracellular delivery beyond HIV-1 surface fusion. Helical stabilization through lactam bridges (Lys(i)–Asp(i + 4)) in EBOV7 enforces α-helical HR2 conformation to trap GP2 prehairpin intermediates, while eboIZN39IQ’s chimeric fusion of natural HR1 to IZ/IQ coiled-coil modules yields 14 nM Kd via stable N-trimer mimicry, linking directly to stapling and macrocyclization for enhanced coiled-coil affinity. Layered engineering in EBOV7 integrates lipidation, PEGylation, stapling, and N/C-terminal extensions into a potent 30-mer, mirroring HIV-1 HR1 strategies for orthogonal optimizations that boost broad-spectrum filovirus inhibition.

## 5. Conclusions and Perspective

Class I viral fusion proteins present a common structural vulnerability across diverse enveloped viruses. Despite sequence divergence, their shared architecture, proteolytic activation, exposure of a hydrophobic fusion element, formation of HR1/HR2 coiled coils, and collapse into a six-helix bundle create opportunities to intercept the fusion cascade. Guided by sequence, high-resolution structures, and computational modeling, peptide inhibitors that mimic HR2 now consistently demonstrate robust in vitro and in vivo activity across HIV-1, RSV, SARS-CoV-2, IAV, NiV, and EBOV. Through structure-guided and chemistry-guided refinements of peptide designs, these peptides have achieved micromolar-to-picomolar potency and significantly improved drug-like properties across Class I viral systems. Serving as strong evidence that the 6HB is a druggable target, multiple peptide inhibitor candidates have progressed to preclinical and clinical evaluation. Several peptide fusion inhibitors have already reached clinical use or advanced clinical evaluation, including enfuvirtide (T20) (HIV-1; FDA-approved) [62,63,65], albuvirtide (HIV-1; Phase II, approved by the Chinese NMPA in June 2018 as a once-weekly injectable fusion inhibitor marketed under the trade name Aikening^®^) [92,191,192], LP-98 (HIV-1, Phase II) [66], YKYY017 (SARS-CoV-2, Phase II) [152], RQ-01 (Phase II) [150] and HY3000 (SARS-CoV-2, approved to conduct clinical investigation by U.S. FDA) [193]. Additional candidates for RSV, IAV, NiV, and EBOV are steadily progressing through preclinical pipelines.

Although Class I viruses are generally thought to share a conserved overall fusion mechanism, individual viruses differ in their responses to distinct peptide engineering strategies, reflecting discrepancies in their fusion protein structures and fusion mechanics. For HIV-1, where the HR1 groove is narrow and the fusion intermediate is short-lived, cholesterol-conjugated HR2 mimetics such as LP-98 [66,104] effectively anchor inhibitors at the membrane and boost local concentration, leading to subpicomolar potency. RSV, by contrast, has a highly metastable prefusion F protein with a deep HR1 pocket, making α-helical stabilization strategies, such as single- or double-stapling (SAH-RSVF_BD_) [131], especially effective for improving protease resistance and prolonging the engagement of the fusion intermediate. For coronaviruses like SARS-CoV-2, N-terminally extended HR2 mimetics and pocket-targeting hydrophobic motifs are particularly effective [147] due to the elongated HR1 coiled-coil and exposed central cavity, while lipopeptide approaches enhance mucosal retention in the respiratory tract [143]. IAV benefits from cyclic or macrocyclic peptide inhibitors that capture conserved structural motifs of HA2 and resist the acidic endosomal environment required for HA activation [168,169]. NiV and EBOV, which rely on endosomal or tightly opposed membrane fusion, respond best to multivalent or lipidated designs that increase avidity and membrane engagement [177,189]. Together, these examples illustrate how matching peptide design features to virus-specific fusion mechanics yields the most potent and durable inhibitors.

The emergence of drug resistance remains a challenge in the clinical application of peptide fusion inhibitors, necessitating a comparative evaluation of how distinct design modalities influence the genetic barrier to viral escape. First-generation linear peptides, such as T20, are susceptible to rapid resistance due to their reliance on flexible, surface-exposed interactions that tolerate amino acid substitutions such as those in the Gly-Ile-Val motif (and adjacent residues) of gp41 without compromising viral fitness [194,195]. In contrast, strategies that target the highly conserved hydrophobic pocket, such as C34-based designs, levy a significantly higher genetic barrier to resistance because escape mutations in this functionally critical region often entail a severe fitness cost to the virus [100,101,194,196]. Beyond sequence selection, chemical modifications play a key role in resistance resilience; lipid conjugation creates a high potency barrier by concentrating the inhibitor at the fusion site, thereby maintaining therapeutic thresholds even against variants with reduced intrinsic binding affinity. Similarly, hydrocarbon stapling and macrocyclization rigidify the peptide into its bioactive helical conformation, effectively overcoming the entropic penalties of binding and retaining potency against escape mutants, such as the RSV K394R variant, that destabilize flexible linear analogs. Multimerization strategies leverage avidity effects, where the cooperative binding of dimeric or trimeric constructs renders single-point mutations insufficient to abolish inhibition. Recent evidence indicates that combinatorial approaches, particularly stapled lipopeptides, offer the most robust protection by effectively “cornering” the virus through simultaneous membrane localization and high-affinity conserved groove engagement [150]. Consequently, to preempt viral evolution, future design paradigms should systematically integrate pocket-specific targeting with membrane anchoring and conformational constraints to limit the evolutionary pathways available for escape, which necessitates continuous monitoring and periodic redesign of peptide inhibitors [39,196].

The development of peptides with sufficient stability against proteolytic degradation and extended half-life in vivo remains challenging despite advances in chemical modification, structural refinements, and residue engineering. Delivery to target tissues and intracellular compartments also poses obstacles, especially for respiratory viruses. From a practical standpoint, peptide drugs often suffer from poor oral bioavailability, which limits their administration routes predominantly to injection or inhalation.

Future directions may focus on overcoming these limitations by integrating computational design, machine learning, and high-resolution structural data to design broadly neutralizing and less resistance-prone peptides. Multivalent and fusion-inhibitory lipopeptides hold promise for improved efficacy and membrane targeting. Oral formulations and novel delivery platforms, such as nanoparticles or intranasal sprays, could broaden clinical applicability. Continued research into viral fusion mechanisms and the identification of conserved targets and conformational plasticity among fusion proteins will further refine inhibitor design, thereby enhancing antiviral strategies across diverse viral families. The core principle is to target multiple conformational checkpoints to achieve broader and more sustained inhibition.

## Figures and Tables

**Table 1:** Characteristics of notable HIV-1 Env fusion inhibitors.

Target Region	Representative Inhibitors	Key Modifications	Potency (IC_50_)	Development Stage	Design Lessons Learned
**gp41-HR1**	T20 [62]	HR2 sequence mimetic	~3–24 nM	FDA-approved	Pocket-filling residues and membrane anchoring Cholesterol critically enhance potency.
	SAH-gp41 [89]	Stapling	~5 nM	in vitro studies	
	CPT31 [106]	Linker conjugation	10–50 pM	Phase I	
	LP-98 [66]	Cholesterol conjugation	sub picomolar	Phase II	

**Table 2:** Characteristics of notable RSV F-directed fusion inhibitors.

Target Regio	Representative Inhibitors	Key Modifications	Potency (IC_50_)	Development Stage	Design Lessons Learned
**F1-HR1**	T118 [123]	HR2 sequence mimetic	~1–10 µM	In vitro studies	Stapling is critical for RSV due to metastable F protein and deep HR1 pocket. Shorter peptides with tight stapling outperform longer unmodified variants.
	SAH-RSVF_BD_ [131]	Double-Stapling	~3–10 nM	Advanced preclinical	
	5-Helix [126]	Multimeric construct	~3.6 µM	Preclinical	

**Table 3:** Characteristics of notable SARS-CoV-2 anti-spike peptide inhibitors.

Target Region	Representative Inhibitors	Key Modifications	Potency (IC_50_)	Development Stage	Design Lessons Learned
**S2-HR1**	EK1 [137]	Optimized H2 mimetic	200 nM—low micromolar	Preclinical	N-terminal HR2 extension (residues 1159–1179) critical for contacts with elongated HR1 groove. Linker rigidity (EAAAK) improves potency.
	P40-LP [147]	N-terminal extension and Lipid conjugation	0.3–2 nM	Preclinical	
	RQ-01 [150]	Stapling and N-terminal lipidation	~3.5–9 nM	Phase II	
	IPB29 [143]	Cholesterol conjugation and linkers addition	~0.5–3 nM	Phase III clinical trials (China)	

**Table 4:** Characteristics of notable Influenza A anti-HA peptide inhibitors.

Target Region	Representative Inhibitors	Key Modifications	Potency (IC_50_)	Development Stage	Design Lessons Learned
**HA2-HR1**	P155-185-Chol [161]	HR2 sequence mimetic and lipid conjugation	~0.2–0.4 µM	in vitro	Macrocyclization design captures conserved HA stalk domain and NPAAs enhance metabolic protease resistance
	Tat-HA2Ec2 [165]	Cell penetrating sequence addition and lipid and linker conjugation	N/A	Preclinical	
	iHA-100 [169]	Macrocyclization (head to side chain thioether bridge)	0.036 µM	Preclinical	
	P7 [166]	Macrocyclization via sidechain-to-tail lactam bridge and non-proteinogenic amino acids (NPAAs) for rigidification	30–70 nM	in vitro	

**Table 5:** Characteristics of notable NiV-F-targeting peptide inhibitors.

Target Region	Representative Inhibitors	Key Modifications	Potency (IC_50_)	Development Stage	Design Lessons Learned
**F1-HR1**	N-PEG-NiV FC2 [174]	N-terminal PEGylation	3–10 nM	in vitro	Chimeric HR1/HR2 constructs (NiV/HeV/HPIV3) reveal heterotypic peptide superiority through optimized interhelical packing. Sequence-specific substitutions (VIKI motif) and lipid conjugation enhance binding avidity.
	N42NiV/HeV(L6) C32HPIV3 [176]	Chimeric fusion core peptide with linker	N/A	in silico	
	VIKI-dPEG4-bisToco [177]	Stabilizing mutations along with PEGylation and lipid conjugation	~1–7 nM	Preclinical	

**Table 6:** Characteristics of notable EBOV GP2 fusion inhibitors.

Target Region	Representative Inhibitors	Key Modifications	Potency (IC_50_)	Development Stage	Design Lessons Learned
**GP2-HR1**	Peptide 1-Chol [187]	Cholesterol conjugation	Low micromolar (<10 µM)	in vitro	Endosomal targeting (Tat or cholesterol) essential due to late-stage fusion compartment. Multilayered modifications (cholesterol, PEG, staples, sequence extension) required for optimal potency
	EBOV7 [189]	Stapling, PEGylation and cholesterol conjugation	N/A	in vitro	
	Tat Ebo [181,189]	Cell-penetrating sequence and linker conjugation	~10 µM	in vitro	

## Data Availability

No new data were created or analyzed in this study. Data sharing is not applicable to this article.

## Abbreviations

The following abbreviations are used in this manuscript:

HIV-1	Human immunodeficiency virus-1
RSV	Respiratory syncytial virus
SARS-CoV-2	Severe acute respiratory syndrome coronavirus 2
IAV	Influenza virus A
NiV	Nipah virus
EBOV	Ebola virus
HR1	Heptad repeat 1/N-terminal heptad repeat/heptad repeat A
HR2	Heptad repeat 2/C-terminal heptad repeat/heptad repeat B
6HB	Six helical bundle
GP	Glycoprotein
HA	Haemagglutinin
PDB	Protein Data Bank
ID	Identifier
SAR	Structure activity refinement
Cryo-EM	Cryo electron microscopy
FDA	Food and Drug Administration
NMPA	National Medical Products Administration
